# Pyridinium 5-nitro­thio­phene-2-carboxyl­ate

**DOI:** 10.1107/S1600536810016089

**Published:** 2010-05-08

**Authors:** Xiao-Yan Yang, Ning Xu, Shan Liu

**Affiliations:** a Department of Chemical Engineering, Nanjing College of Chemical Technology, Nanjing 210048, People’s Republic of China

## Abstract

The anion of the title compound, C_5_H_6_N^+^·C_5_H_2_NO_4_S^−^, is approximately planar, with the carboxyl­ate and nitro group planes forming dihedral angles of 7.5 (3) and 3.5 (3)°, respectively, with the thio­phene ring. In the crystal structure, the cations and anions are linked into a two-dimensional network parallel to (011) by N—H⋯O and C—H⋯O hydrogen bonds.

## Related literature

For the uses of 5-nitro­thio­phene-2-carboxylic acid, see: Cao *et al.* (2003 ▶). For the synthesis, see: Marques *et al.* (2002 ▶). For bond-length data, see: Allen *et al.* (1987 ▶).
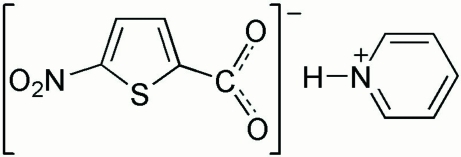


## Experimental

### 

#### Crystal data

C_5_H_6_N^+^·C_5_H_2_NO_4_S^−^
*M* *_r_* = 252.24
Triclinic, 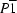

*a* = 6.0940 (12) Å
*b* = 7.3390 (15) Å
*c* = 13.296 (3) Å
α = 77.30 (3)°
β = 81.52 (3)°
γ = 71.00 (3)°
*V* = 546.6 (2) Å^3^
*Z* = 2
Mo *K*α radiation
μ = 0.30 mm^−1^
*T* = 298 K
0.30 × 0.20 × 0.10 mm

#### Data collection

Enraf–Nonius CAD-4 diffractometer
Absorption correction: ψ scan (North *et al.*, 1968 ▶) *T*_min_ = 0.915, *T*_max_ = 0.971
2194 measured reflections
1990 independent reflections
1657 reflections with *I* > 2σ(*I*)
*R*_int_ = 0.019
3 standard reflections every 200 reflections  intensity decay: 1%

#### Refinement

*R*[*F* ^2^ > 2σ(*F* ^2^)] = 0.039
*wR*(*F* ^2^) = 0.080
*S* = 1.00
1990 reflections
155 parameters
H-atom parameters constrained
Δρ_max_ = 0.25 e Å^−3^
Δρ_min_ = −0.22 e Å^−3^

### 

Data collection: *CAD-4 Software* (Enraf–Nonius, 1985 ▶); cell refinement: *CAD-4 Software*; data reduction: *XCAD4* (Harms & Wocadlo, 1995 ▶); program(s) used to solve structure: *SHELXS97* (Sheldrick, 2008 ▶); program(s) used to refine structure: *SHELXL97* (Sheldrick, 2008 ▶); molecular graphics: *SHELXTL* (Sheldrick, 2008 ▶); software used to prepare material for publication: *SHELXTL*.

## Supplementary Material

Crystal structure: contains datablocks I, global. DOI: 10.1107/S1600536810016089/ci5088sup1.cif

Structure factors: contains datablocks I. DOI: 10.1107/S1600536810016089/ci5088Isup2.hkl

Additional supplementary materials:  crystallographic information; 3D view; checkCIF report

## Figures and Tables

**Table 1:** Hydrogen-bond geometry (Å, °)

*D*—H⋯*A*	*D*—H	H⋯*A*	*D*⋯*A*	*D*—H⋯*A*
N1—H6⋯O2	0.86	1.74	2.594 (3)	176
C3—H3⋯O1	0.93	2.49	3.156 (3)	129
C1—H1⋯O3^i^	0.93	2.55	3.254 (3)	133
C4—H4⋯O1^ii^	0.93	2.44	3.210 (3)	141

Symmetry codes: (i) 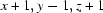; (ii) 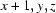.

**Fig. 1.**
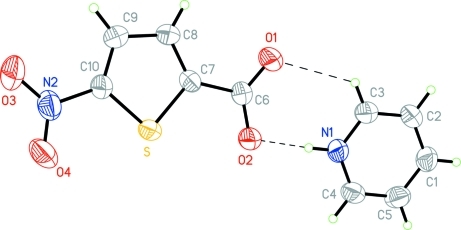
The asymmetric unit of the title compound, with the atom-numbering scheme. Displacement ellipsoids are drawn at the 50% probability level. Hydrogen bonds are shown as dashed lines.

**Fig. 2.**
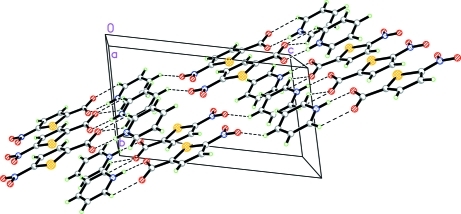
Crystal packing of the title compound. hydrogen bonds are shown as dashed lines.

## supplementary crystallographic information

### Comment

5-Nitrothiophene-2-carboxylic acid is an important intermediate used to synthesize raltitrexed (trade name Tomudex), an antimetabolite drug used in cancer chemotherapy (Cao *et al.*, 2003). We report here the crystal structure of the title compound (Fig. 1).

Bond lengths and angles in both cation and anion are within normal ranges (Allen *et al.*, 1987). The anion is approximately planar; the C6/O1/O2 and N2/O3/O4 planes form dihedral angles of 7.5 (3)° and 3.5 (3)°, respectively, with the thiophene ring. In the crystal structure, the cations and anions are linked into a two-dimensional network (Fig. 2) by N—H···O and C—H···O hydrogen bonds.

### Experimental

5-Nitrothiophene-2-carboxylic acid was prepared by the method reported in literature (Marques *et al.*, 2002). Single crystals were obtained by dissolving 5-nitrothiophene-2-carboxylic acid (0.5 g, 2.89 mmol) in pyridine (50 ml) and evaporating the solvent slowly at room temperature for about 20 d.

### Refinement

After checking their presence in the difference map, all the H atoms were positioned geometrically [N—H = 0.86 and C—H = 0.93 Å] and constrained to ride on their parent atoms, with U_iso_(H) = 1.2U_eq_(C,N).

### Crystal data

**Table:**

C_5_H_6_N^+^·C_5_H_2_NO_4_S^−^	*Z* = 2
*M**_r_* = 252.24	*F*(000) = 260
Triclinic, *P*1	*D*_x_ = 1.533 Mg m^−^^3^
Hall symbol: -P 1	Mo *K*α radiation, λ = 0.71073 Å
*a* = 6.0940 (12) Å	Cell parameters from 25 reflections
*b* = 7.3390 (15) Å	θ = 10–13°
*c* = 13.296 (3) Å	µ = 0.30 mm^−^^1^
α = 77.30 (3)°	*T* = 298 K
β = 81.52 (3)°	Block, colourless
γ = 71.00 (3)°	0.30 × 0.20 × 0.10 mm
*V* = 546.6 (2) Å^3^	

### Data collection

**Table:**

Enraf–Nonius CAD-4 diffractometer	1657 reflections with *I* > 2σ(*I*)
Radiation source: fine-focus sealed tube	*R*_int_ = 0.019
graphite	θ_max_ = 25.3°, θ_min_ = 1.6°
ω/2θ scans	*h* = 0→7
Absorption correction: ψ scan (North *et al.*, 1968)	*k* = −8→8
*T*_min_ = 0.915, *T*_max_ = 0.971	*l* = −15→15
2194 measured reflections	3 standard reflections every 200 reflections
1990 independent reflections	intensity decay: 1%

### Refinement

**Table:**

Refinement on *F*^2^	Secondary atom site location: difference Fourier map
Least-squares matrix: full	Hydrogen site location: inferred from neighbouring sites
*R*[*F*^2^ > 2σ(*F*^2^)] = 0.039	H-atom parameters constrained
*wR*(*F*^2^) = 0.080	*w* = 1/[σ^2^(*F*_o_^2^) + (0.01*P*)^2^ + 0.48*P*] where *P* = (*F*_o_^2^ + 2*F*_c_^2^)/3
*S* = 1.00	(Δ/σ)_max_ = 0.001
1990 reflections	Δρ_max_ = 0.25 e Å^−^^3^
155 parameters	Δρ_min_ = −0.22 e Å^−^^3^
0 restraints	Extinction correction: *SHELXL97* (Sheldrick, 2008), Fc^*^=kFc[1+0.001xFc^2^λ^3^/sin(2θ)]^-1/4^
Primary atom site location: structure-invariant direct methods	Extinction coefficient: 0.0313 (18)

### Special details

**Table:**

Geometry. All esds (except the esd in the dihedral angle between two l.s. planes) are estimated using the full covariance matrix. The cell esds are taken into account individually in the estimation of esds in distances, angles and torsion angles; correlations between esds in cell parameters are only used when they are defined by crystal symmetry. An approximate (isotropic) treatment of cell esds is used for estimating esds involving l.s. planes.
Refinement. Refinement of *F*^2^ against ALL reflections. The weighted *R*-factor wR and goodness of fit *S* are based on *F*^2^, conventional *R*-factors *R* are based on *F*, with *F* set to zero for negative *F*^2^. The threshold expression of *F*^2^ > σ(*F*^2^) is used only for calculating *R*-factors(gt) etc. and is not relevant to the choice of reflections for refinement. *R*-factors based on *F*^2^ are statistically about twice as large as those based on *F*, and *R*- factors based on ALL data will be even larger.

### Fractional atomic coordinates and isotropic or equivalent isotropic displacement parameters (Å^2^)

**Table:**

	*x*	*y*	*z*	*U*_iso_*/*U*_eq_	
S	0.14233 (10)	0.12191 (9)	0.65371 (4)	0.04471 (19)	
O1	−0.0782 (3)	−0.1820 (3)	0.90222 (13)	0.0592 (5)	
O2	0.2305 (3)	−0.0690 (3)	0.86414 (12)	0.0534 (5)	
O3	−0.1575 (4)	0.3584 (3)	0.40288 (14)	0.0753 (6)	
O4	0.1837 (3)	0.3262 (3)	0.44510 (14)	0.0691 (6)	
N2	−0.0093 (4)	0.3011 (3)	0.46450 (15)	0.0538 (5)	
C6	0.0413 (4)	−0.0881 (3)	0.84465 (17)	0.0419 (5)	
C7	−0.0384 (4)	0.0149 (3)	0.74043 (16)	0.0375 (5)	
C8	−0.2423 (4)	0.0392 (3)	0.70084 (17)	0.0438 (6)	
H8	−0.3598	−0.0095	0.7374	0.053*	
C9	−0.2565 (4)	0.1453 (4)	0.59945 (18)	0.0469 (6)	
H9	−0.3833	0.1760	0.5608	0.056*	
C10	−0.0599 (4)	0.1972 (3)	0.56517 (17)	0.0427 (5)	
N1	0.3544 (3)	−0.2797 (3)	1.04230 (14)	0.0437 (5)	
H6	0.3164	−0.2065	0.9836	0.052*	
C1	0.4731 (4)	−0.5108 (4)	1.22741 (19)	0.0518 (6)	
H1	0.5143	−0.5902	1.2908	0.062*	
C2	0.2816 (4)	−0.5107 (4)	1.18469 (19)	0.0521 (6)	
H2	0.1911	−0.5896	1.2189	0.063*	
C3	0.2254 (4)	−0.3937 (4)	1.09159 (18)	0.0481 (6)	
H3	0.0960	−0.3932	1.0620	0.058*	
C4	0.5408 (4)	−0.2773 (4)	1.08249 (18)	0.0496 (6)	
H4	0.6285	−0.1969	1.0470	0.060*	
C5	0.6035 (4)	−0.3923 (4)	1.17561 (19)	0.0529 (6)	
H5	0.7335	−0.3906	1.2038	0.063*	

### Atomic displacement parameters (Å^2^)

**Table:**

	*U*^11^	*U*^22^	*U*^33^	*U*^12^	*U*^13^	*U*^23^
S	0.0397 (3)	0.0594 (4)	0.0383 (3)	−0.0262 (3)	−0.0043 (2)	0.0019 (3)
O1	0.0587 (11)	0.0758 (13)	0.0452 (10)	−0.0380 (10)	−0.0053 (8)	0.0121 (9)
O2	0.0500 (10)	0.0674 (12)	0.0460 (10)	−0.0303 (9)	−0.0133 (8)	0.0084 (8)
O3	0.0928 (15)	0.0900 (16)	0.0461 (11)	−0.0385 (12)	−0.0276 (11)	0.0126 (10)
O4	0.0766 (13)	0.0872 (15)	0.0508 (11)	−0.0496 (12)	0.0058 (10)	0.0027 (10)
N2	0.0697 (15)	0.0555 (14)	0.0393 (11)	−0.0277 (12)	−0.0060 (11)	−0.0010 (10)
C6	0.0437 (13)	0.0418 (13)	0.0382 (12)	−0.0141 (11)	−0.0038 (10)	−0.0016 (10)
C7	0.0373 (12)	0.0384 (12)	0.0370 (12)	−0.0159 (10)	0.0013 (9)	−0.0035 (9)
C8	0.0355 (12)	0.0505 (14)	0.0459 (13)	−0.0190 (11)	−0.0012 (10)	−0.0024 (11)
C9	0.0389 (13)	0.0553 (15)	0.0485 (14)	−0.0182 (11)	−0.0105 (10)	−0.0030 (11)
C10	0.0470 (13)	0.0473 (14)	0.0340 (12)	−0.0180 (11)	−0.0057 (10)	−0.0009 (10)
N1	0.0438 (11)	0.0481 (12)	0.0353 (10)	−0.0144 (9)	−0.0036 (8)	0.0016 (9)
C1	0.0519 (15)	0.0557 (16)	0.0409 (13)	−0.0138 (12)	−0.0077 (11)	0.0037 (11)
C2	0.0538 (15)	0.0504 (15)	0.0527 (15)	−0.0254 (12)	−0.0040 (12)	0.0041 (12)
C3	0.0437 (13)	0.0541 (15)	0.0491 (14)	−0.0214 (12)	−0.0086 (11)	−0.0014 (12)
C4	0.0424 (13)	0.0602 (16)	0.0479 (14)	−0.0237 (12)	0.0007 (11)	−0.0040 (12)
C5	0.0417 (14)	0.0702 (18)	0.0484 (14)	−0.0199 (13)	−0.0078 (11)	−0.0074 (13)

### Geometric parameters (Å, °)

**Table:**

S—C10	1.705 (2)	N1—C4	1.331 (3)
S—C7	1.717 (2)	N1—C3	1.335 (3)
O1—C6	1.233 (3)	N1—H6	0.86
O2—C6	1.275 (3)	C1—C2	1.371 (3)
O3—N2	1.218 (3)	C1—C5	1.374 (3)
O4—N2	1.230 (3)	C1—H1	0.93
N2—C10	1.432 (3)	C2—C3	1.361 (3)
C6—C7	1.492 (3)	C2—H2	0.93
C7—C8	1.364 (3)	C3—H3	0.93
C8—C9	1.400 (3)	C4—C5	1.364 (3)
C8—H8	0.93	C4—H4	0.93
C9—C10	1.360 (3)	C5—H5	0.93
C9—H9	0.93		
			
C10—S—C7	89.85 (11)	C4—N1—C3	121.1 (2)
O3—N2—O4	123.7 (2)	C4—N1—H6	119.4
O3—N2—C10	118.7 (2)	C3—N1—H6	119.4
O4—N2—C10	117.6 (2)	C2—C1—C5	119.3 (2)
O1—C6—O2	126.8 (2)	C2—C1—H1	120.3
O1—C6—C7	118.1 (2)	C5—C1—H1	120.3
O2—C6—C7	115.03 (19)	C3—C2—C1	119.2 (2)
C8—C7—C6	129.1 (2)	C3—C2—H2	120.4
C8—C7—S	112.21 (16)	C1—C2—H2	120.4
C6—C7—S	118.70 (16)	N1—C3—C2	120.6 (2)
C7—C8—C9	113.0 (2)	N1—C3—H3	119.7
C7—C8—H8	123.5	C2—C3—H3	119.7
C9—C8—H8	123.5	N1—C4—C5	120.2 (2)
C10—C9—C8	110.8 (2)	N1—C4—H4	119.9
C10—C9—H9	124.6	C5—C4—H4	119.9
C8—C9—H9	124.6	C4—C5—C1	119.5 (2)
C9—C10—N2	126.6 (2)	C4—C5—H5	120.3
C9—C10—S	114.08 (17)	C1—C5—H5	120.3
N2—C10—S	119.34 (17)		
			
O1—C6—C7—C8	7.6 (4)	O4—N2—C10—C9	−176.1 (2)
O2—C6—C7—C8	−172.7 (2)	O3—N2—C10—S	−178.2 (2)
O1—C6—C7—S	−172.45 (18)	O4—N2—C10—S	2.3 (3)
O2—C6—C7—S	7.3 (3)	C7—S—C10—C9	0.4 (2)
C10—S—C7—C8	−0.19 (19)	C7—S—C10—N2	−178.2 (2)
C10—S—C7—C6	179.87 (19)	C5—C1—C2—C3	0.3 (4)
C6—C7—C8—C9	179.9 (2)	C4—N1—C3—C2	0.1 (4)
S—C7—C8—C9	−0.1 (3)	C1—C2—C3—N1	−0.2 (4)
C7—C8—C9—C10	0.3 (3)	C3—N1—C4—C5	0.0 (4)
C8—C9—C10—N2	178.0 (2)	N1—C4—C5—C1	0.1 (4)
C8—C9—C10—S	−0.5 (3)	C2—C1—C5—C4	−0.2 (4)
O3—N2—C10—C9	3.4 (4)		

### Hydrogen-bond geometry (Å, °)

**Table:**

*D*—H···*A*	*D*—H	H···*A*	*D*···*A*	*D*—H···*A*
N1—H6···O2	0.86	1.74	2.594 (3)	176
C3—H3···O1	0.93	2.49	3.156 (3)	129
C1—H1···O3^i^	0.93	2.55	3.254 (3)	133
C4—H4···O1^ii^	0.93	2.44	3.210 (3)	141

Symmetry codes: (i) *x*+1, *y*−1, *z*+1; (ii) *x*+1, *y*, *z*.
